# Dose Escalation and Co-therapy Intensification Between Etanercept, Adalimumab, and Infliximab: The CADURA Study

**DOI:** 10.2174/1874312901711010123

**Published:** 2017-10-24

**Authors:** Carter Thorne, Gilles Boire, Andrew Chow, Kirsten Garces, Fang Liu, Melanie Poulin-Costello, Valery Walker, Boulos Haraoui

**Affiliations:** 1 The Arthritis Program Research Group, Southlake Regional Health Centre, c/o 43 Lundy’s Lane, Newmarket, ON, L3Y 3R7, Canada; 2 Centre Hospitalier Universitaire de Sherbrooke (CIUSSS de l’Estrie-CHUS), Université de Sherbrooke, Sherbrooke, QC, Canada; 3 Credit Valley Rheumatology, Mississauga, ON, Canada; 4 Amgen Canada Inc., Mississauga, ON, Canada; 5 Optum, 5500 North Service Road, Suite 501, Burlington, ON, L7L 6W6, Canada; 6 Institut de Rhumatologie de Montreal, Montreal, QC, Canada

**Keywords:** Rheumatoid arthritis, Etanercept, Adalimumab, Infliximab, Dose escalation, Intensification

## Abstract

**Objective:**

To compare anti-TNF dose escalation, DMARD and/or glucocorticoid intensification, switches to another biologic, and drug and drug-related costs over 12 and 18 months for rheumatoid arthritis (RA) patients initiating etanercept (ETN), adalimumab (ADA), or infliximab (IFX) in routine clinical practice across Canada.

**Methods:**

A retrospective chart review of biologic-naïve adult RA patients newly initiating ADA, ETN, or IFX between January 01, 2006 and December 31, 2012 from 11 practices across Canada.

**Results:**

There were 314 patients in the 12-month analysis and 217 in the 18-month analysis. No dose escalation occurred with ETN over 12 and 18 months versus 38% and 32% for IFX (p<0.001) and 2% and 2% for ADA (p=0.199, p=0.218). Over 18 months, dose escalation and/or DMARD and/or glucocorticoid intensification was less frequent among ETN (16%) versus IFX (44%, p=0.005) and ADA (34%, p=0.004). By 18 months, 22% of patients initiating ADA had switched to another biologic compared with 6% of ETN patients (p=0.001).

Patients initiating ETN had lower total (drug and drug-related) costs over 12 and 18 months compared to IFX, and no difference compared to ADA when adjusted for potential confounders. Patients with dose escalation had higher costs compared to those with no dose escalation.

**Conclusion:**

Physicians were more likely to escalate the dose of IFX, but optimize co-therapy with ADA and ETN. ETN patients had no dose escalation and were less likely to have DMARD and/or glucocorticoid intensification than ADA patients. ETN-treated patients had lower costs compared to IFX patients.

## 1 INTRODUCTION

Rheumatoid arthritis (RA), a progressive disease requiring lifelong treatment, affects approximately 1% of the Canadian population [1]. The goal of RA therapy is to reduce disease activity, and ultimately, provide disease remission. The effectiveness of current therapies, including disease-modifying antirheumatic drugs (DMARDs), anti-tumor necrosis factor (TNF) agents, and other biologic drugs have made these goals achievable. The most commonly prescribed anti-TNF agents, etanercept (ETN), infliximab (IFX), and adalimumab (ADA), have proven effective at reducing signs and symptoms and slowing progression of RA [2].

In addition to differences in method of administration and dosing schedule, ETN, IFX, and ADA have important molecular differences that may affect immunogenicity and long-term clinical efficacy [3]. ETN is a recombinant human soluble TNF-receptor protein, while both ADA and IFX are anti-TNF monoclonal antibodies. Studies have shown that patients receiving either ADA or IFX developed neutralizing antibodies against the drugs, contributing to a loss of therapeutic response [4-8]. Neutralizing antibodies were detected in 33% of patients receiving IFX [7] and 28% of patients on ADA [8]. Due to inadequate therapeutic response, clinicians often escalate or intensify the dose of the drug or switch to another biologic agent [7-10]. Dose escalation increases drug treatment costs [11-14], patient inconvenience, and risk of adverse events (*e.g.*, infusion reactions, infections) [15-17], without necessarily offering additional clinical benefit [11, 18-20]. 

European and US studies have documented higher rates of dose escalation in patients receiving IFX and ADA compared to ETN [11, 12, 18, 20-27]. The DART study (Drug utilization and dosing patterns Assessment: A Retrospective observational study of subjects Treated for rheumatoid arthritis) [27], encompassing patients in 5 European countries, showed the proportion with dose escalation necessary to maintain a clinical response was significantly higher in patients receiving IFX or ADA compared to patients receiving ETN over 12 months. Similarly, patients requiring dose escalation and/or adding/intensifying DMARDs or glucocorticoids over 12 months was higher in patients receiving IFX or ADA than ETN. Total annual medical costs were higher in patients receiving ADA compared to ETN, and dose escalation resulted in higher costs compared to no dose escalation for patients receiving ADA and IFX, but not for ETN [11]. DART II [18], a US chart review and claims-based study, found lower dose escalation rates and anti-TNF costs with ETN compared to ADA and IFX.

While comparisons between IFX, ADA, and ETN have been documented in several studies, there is a lack of results from a Canadian clinical practice population. This study was conducted using real-world data from a Canadian setting to estimate dose escalation, co-therapy intensification, discontinuation, switching patterns, and treatment costs over 12 and 18 months for RA patients initiating ETN, ADA, or IFX.

## 2 MATERIALS AND METHODS

### 2.1 Study Design and Data Source

This study was a retrospective chart review of biologic-naïve, adult patients who newly initiated ADA, ETN, or IFX between January 01, 2006 and December 31, 2012. The index date was defined as the date of treatment initiation (or prescription date if unknown) for the index medication, and the index anti-TNF was the first anti-TNF initiated during the study period. Medical records were obtained from 11 rheumatology clinics from private practice and teaching hospitals across Canada, representing 5 provinces (Ontario, Quebec, New Brunswick, Saskatchewan, and Newfoundland). Medical charts were abstracted 3 months prior to the index date and 12 and 18 months following and including the index date. Charts were selected based on reverse chronological order starting with patients treated with etanercept, adalimumab, or infliximab on December 31, 2011 and going backwards in time until enough charts were identified. The protocol and a waiver of patient consent were approved by Institutional Review Board Services and separate approvals were obtained at the facility level as needed. Confidentiality of all data was preserved.

### 2.2 Patient Identification

RA patients (≥18 years old) treated continuously with an initial biologic for at least 6 months following the index date who had at least 3 physician visits during the first year following the index date (with at least 1 visit in months 9-15 for the 12-month analysis and at least 1 visit in between months 15-21 for the 18-month analysis) were eligible; those with any prior biologic therapy or a concurrent diagnosis of Crohn’s disease, ulcerative colitis, juvenile idiopathic arthritis, systemic lupus erythematosus, psoriasis, psoriatic arthritis, or ankylosing spondylitis were excluded, as were patients involved in any clinical trial or receiving any investigational drug within 28 days of the index date or throughout the study.

### 2.3 Study Outcomes

The primary outcome was dose escalation over 12 months follow-up, defined as the first occurrence of any upward adjustment in dose or dosing frequency of the index anti-TNF from the label/indicated dose and dose frequency (ETN 25 mg twice weekly or 50 mg once weekly, ADA 40 mg once every other week, or IFX 3 mg/kg every 8 weeks after the third infusion). Dose escalation was also measured over 18 months. Alternative definitions of dose escalation included the mean dose and dosing frequency and the last dose of the index anti-TNF exceeding the label/indicated dose and frequency. DMARD dose increases for titration and IFX dose increases due to weight gain were not considered escalations. The proportion of patients who switched to a different biologic, discontinued but did not switch, or intensified DMARD or glucocorticoid or both were also measured over 12 and 18 months. DMARD intensification was defined as any upward adjustment in dose or frequency, addition of a new DMARD, or a switch from an oral to subcutaneous or intramuscular (IM) DMARD 3 months after initiation of the anti-TNF. Similarly, glucocorticoid intensification was defined as an increase in dose or frequency, addition of a new glucocorticoid, or any IM or intra-articular (IA) glucocorticoid injections 3 months after initiation of the anti-TNF. Dose de-escalation was measured over 12 and 18 months and was defined as a decrease in initiating dose or a reduction in dosing frequency. The time to first dose escalation of the index anti-TNF was measured. The magnitude of dose escalation was calculated as the percent change from baseline dose to dose at first escalation. The baseline dose for IFX was the dose after the third infusion.

Drug and drug-related costs over the full 12 and 18 months were examined, including time after patients discontinued or switched index medication for those that did. All drug and drug-related costs were obtained from the province of Ontario and are reported in Canadian dollars. If 2015 costs were not available, earlier costs were adjusted to 2014 [28]. Medications were priced using the best available price for drugs listed on the Ontario Drug Benefit Formulary [29]. Drugs not listed on the formulary were obtained from the Ontario Exceptional Access Program [30]. An 8% markup was used for all prescription drug costs, not including the dispensing fee [31]. The cost of ETN was $195.31 for 25 mg and $390.74 for 50 mg, ADA $740.36 for 40 mg, and IFX $987.56 per 100 mg. A dispensing fee of $8.83 was added as applicable [32]. Healthcare professional fees for intravenous (IV) infusions of biologics and IM or IA injections of glucocorticoids and DMARDs were obtained from the Ontario Schedule of Benefits for Physician Services [33]. The administrative cost for each IV administration episode of IFX was $297.02 (based on $187 per hour [34] for 1.5 hours per infusion). No administration costs were included for subcutaneous injections, as they were assumed to be patient administered. Additional costs (*e.g.*, saline, saline bags) were obtained from Surgo Surgical Supply [35]. Total drug costs were the sum of the direct drug costs and drug administration costs.

### 2.4 Statistical Analysis

A chi-square test (two-sided, α=0.05) was used to establish sample size based on the rates of dose escalation obtained from published literature to detect differences in the proportion of escalators. A total of 329 patients (137 ETN, 137 ADA, and 55 IFX) were expected to have 80% power to detect expected differences in escalation between ETN and ADA cohorts and 99% power to detect differences in escalation between ETN and IFX cohorts. To maintain the same power for the 18-month analysis with different estimates of escalation, the total increased to 445 patients (195 ETN, 195 ADA, and 55 IFX).

All statistical tests, unless otherwise noted, were 2-sided tests performed at a significance level of 0.05. For the primary analysis of no difference between treatment cohorts, sequential testing with fixed sequences was employed to preserve the family-wise error rate at α=0.05. All other p-values are descriptive. The null hypothesis of no difference in the proportion of patients with dose escalation between the ETN and IFX cohorts was tested using a chi-square test. If the null hypothesis was rejected, a conditional pair-wise comparison was implemented for the null hypothesis of no difference between ETN and ADA. Differences in other measures between ETN and IFX and between ETN and ADA were examined using chi-square tests (categorical variables) and t-tests (continuous variables). Subgroup analyses examined clinical and demographic factors related to dose escalation over 12 months with differences assessed by chi-square test. A generalized linear model (GLM) using gamma distribution with log link function was used to compare mean costs by cohort over 12 and 18 months. The model adjusted for Quan-Charlson comorbidity index score, age, duration of RA, gender, medication insurance, prior 3-month DMARD use, and initiation of anti-TNF monotherapy versus concomitant DMARD. Those with missing RA duration data were excluded from the model. A sensitivity analysis was conducted including patients with missing RA duration, setting the duration to 0-10 years, which matched the duration for 65% of patients who had duration data.

Kaplan-Meier curves were created to describe first dose escalation over time among the 3 cohorts without adjusting for confounders.

## 3 RESULTS

### 3.1 Patients

**Fig. (1):**
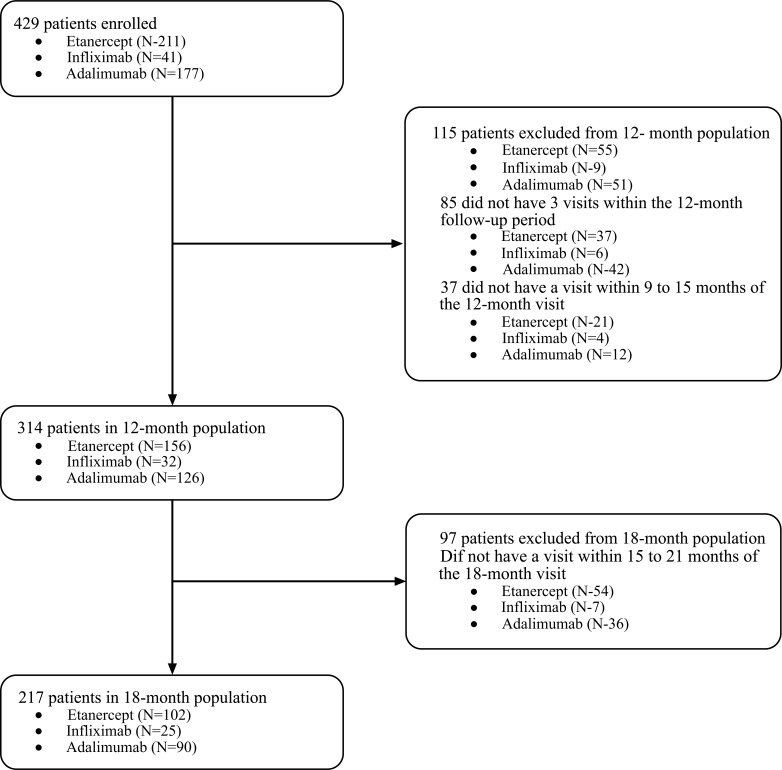
Study sample selection flowchart.

The final sample included 314 patients in the 12-months analysis and 217 who remained in the 18-month analysis (Fig. **1**).

The three cohorts were balanced in regards to demographic and clinical characteristics (Table **1**).

The population was predominantly female (76.8%), with a mean age of 56.3 years (range 21.0 to 90.0 years) and average disease duration of 9.0 years (range 2.0 to 26.0 years). The majority were receiving a concomitant DMARD (77.1%) at the time of anti-TNF initiation. More patients initiating ADA were taking a concomitant DMARD three months prior to (53.2%) and at anti-TNF initiation (83.3%) than patients initiating ETN [40.4% (p=0.041) and 72.4% (p=0.033), respectively]. Comparing patients initiating IFX to ETN, there were no differences in DMARDs three months prior to (31.3%; p=0.427) and at anti-TNF initiation (75.0%; p=0.833).

### 3.2 Anti-TNF Dose Escalation

No dose escalation was observed for ETN (95% confidence interval (CI) 0.0%, 2.3%), versus 37.5% (95% CI 21.1%, 56.3%) for IFX (p<0.001) and 1.6% (95% CI 0.2%, 5.6%) for ADA (p=0.199) over 12 months. (Table **2**, Supplementary Figure A). Over 18 months, no dose escalation was observed for ETN (95% confidence interval (CI) 0.0%, 3.6%), versus 32.0% (95% CI 15.0%, 53.5%) for IFX (p<0.001) and 2.2% (95% CI 0.3%, 7.8%) for ADA (p=0.218).

Results were the same using the two alternative definitions, mean dose escalation and last dose escalation (data not shown). Subgroups provided no evidence of any differences of dose escalation patterns (data not shown).

### 3.3 Anti-TNF Dose Escalation/Co-Therapy Intensification

More IFX patients (40.6%) required dose escalation and/or intensification with a DMARD over 12 months compared to ETN (10.9%; p<0.001) (Table **2**). While 14.3% of patients on ADA required dose escalation and/or intensification with a DMARD, the difference from ETN was not statistically significant (p=0.468). A higher proportion of patients receiving IFX (46.9%) or ADA (27.8%) required dose escalation and/or intensification with a DMARD and/or glucocorticoids over 12 months compared to patients initiating ETN (16.0%) (p<0.001 and p=0.019 respectively). Results over 18 months were similar.

### 3.4 Switching/Discontinuation

Discontinuation of index anti-TNF was similar between ETN and IFX and ETN and ADA over 12 months; however, by 18 months, fewer patients initiating ETN discontinued and switched to another biologic (5.9%) compared to ADA (22.2%; p=0.001) (Table **2**).

### 3.5 Dose De-Escalation

Over 12 months, dose de-escalation was observed in 0.6% of patients who initiated ETN and in 0.0% of patients who initiated ADA or IFX (p=1.000). Over 18 months, slightly more ETN patients had evidence of dose de-escalation (2.0%) compared with ADA (1.1%) and IFX (0.0%), but the differences were not significant (p=1.000).

### 3.6 Magnitude and Time to Dose Escalation

Among patients requiring dose escalation, the mean dose of IFX, converted to a dose every 8 weeks, increased from 3.4 mg/kg after the third infusion to 4.5 mg/kg at first escalation, for a mean change of 32.9% (n=12). The mean dose of ADA increased from 40 mg every other week to 40 mg every week, for a mean change of 100% (n=2).

**Fig. (2):**
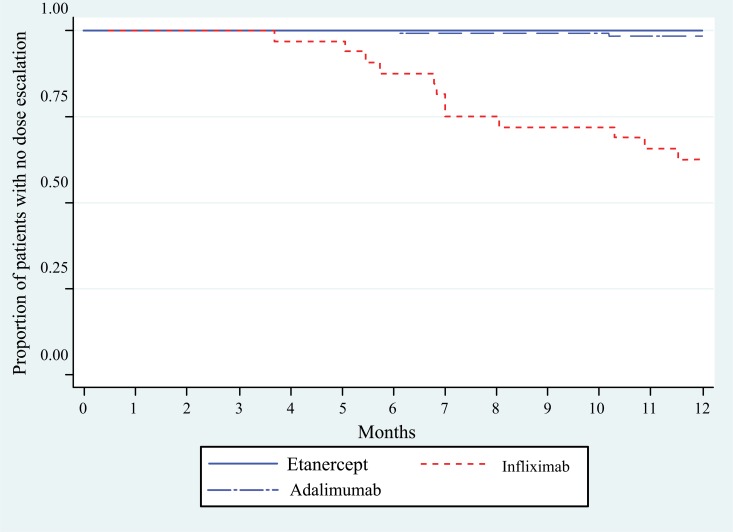
Time to dose escalation over 12 months^1^.

^1^The time to first dose escalation was set as the full follow-up period for patients who did not have a dose escalation (right censored).

Patients initiating IFX began dose escalation between 4 and 7 months (Fig. **2**). The mean (standard deviation) time to dose escalation within the subset of patients who dose escalated was 224 days (74 days) for IFX (n=12) and 248 days (88 days) for ADA (n=2). No further dose escalations were observed in the 18-month follow-up (data not shown).

### 3.7 Costs of Treatment

**Fig. (3):**
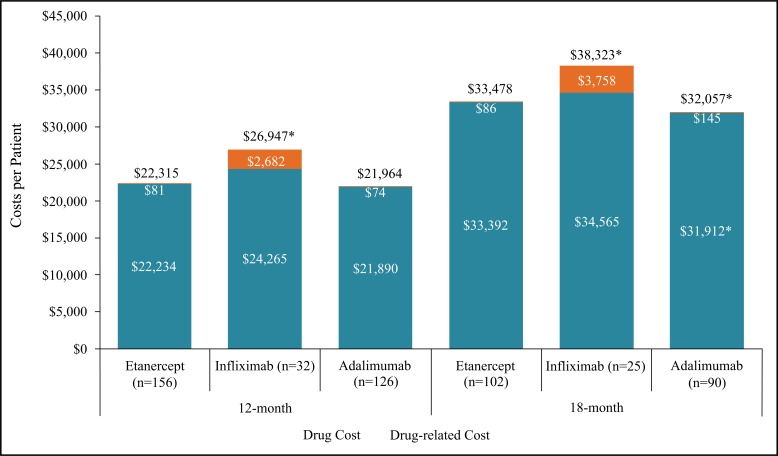
Mean cost (2014/2015 Canadian Dollars) per patient by drug and drug-related costs^1^ over 12 and 18 months.

Over 12 months, mean drug costs (anti-TNFs, switched biologics, DMARDs, and glucocorticoids) were similar between patients initiating ETN ($22,234) versus those initiating IFX ($24,265, p=0.09) or ADA ($21,890, p=0.156). However, due mainly to the cost of IV infusions, the mean drug-related cost (health care professional fees for IV infusions, IM or IA injections) was higher in IFX patients ($2,682) than patients on ETN ($81, p<0.001) (Fig. **3**). Over 18 months, drug costs for patients initiating ETN ($33,392) were higher than for patients initiating ADA ($31,912, p=0.009) (Fig. **3**). The mean cost of switched biologics was higher for ADA initiators ($2,585) versus ETN ($570, p=0.005), as was the mean cost of DMARDs ($767 in ADA initiators versus $540 in ETN initiators, p=0.017); the mean index anti-TNF costs were $3,727 lower in ADA initiators ($28,529) compared to ETN initiators ($32,256, p<0.001).

The mean total unadjusted costs over 12 months were lower in ETN initiators ($22,315) compared to those initiating IFX ($26,947, p<0.001), but not significantly different from patients initiating ADA ($21,964, p=0.156) (Fig. **3**). Over 18 months, total costs in ETN initiators ($33,478) remained lower than IFX ($38,323, p=0.044), but were higher than patients initiating ADA ($32,057, p=0.012). When the mean total costs were adjusted for confounders, the difference between patients initiating ETN and ADA over 18 months was no longer statistically significant (p=0.09 base case, p=0.059 sensitivity analysis) (Supplementary Table **B**).

Compared to no DMARD use in the 3 months prior to index medication, patients with more than one DMARD had higher 12-month mean total costs (cost ratio 1.082, p=0.007, sensitivity analysis) and higher 18-month total costs (cost ratio 1.093, p=0.045, sensitivity analysis) (Supplementary Tables **A** and **B**). Additionally, a one score increase in Charlson comorbidity score was associated with lower 18-month mean total costs (cost ratio 0.927, p=0.001, base-case analysis, cost ratio 0.941, p=0.005, sensitivity analysis) and compared to patients with government insurance (n=159), patients with no insurance (n=12) had lower 12-month mean total costs (cost ratio 0.899, p=0.004, sensitivity analysis).

Mean total costs over 12 months were $9,805 higher among dose escalators versus non-escalators taking IFX (p<0.001) (Table **3**).

Over 18 months, the mean total costs were $16,126 and $13,599 higher in dose escalators versus non-escalators for patients initiating IFX (p<0.001) and ADA, respectively (p<0.001).

## 4 DISCUSSION

Our results indicate that Canadian physicians are more likely to escalate the dose of IFX (12 months, 38%; 18 months, 32%), but not ADA (12 and 18 months, 2%) compared to ETN (12 and 18 months, 0%). Dose escalation of IFX was lower over 18 months compared to 12 months due to fewer patients in the 18 month cohort compared to the 12 month cohort (217 *vs*. 314) and that some of the patients with a dose escalation over 12 months were not included in the 18 month follow-up sample. Recent studies have reported higher dose escalation rates in both IFX and ADA treated patients compared to those treated with ETN [11, 18, 21, 27]. The DART study, from which our clinical measures were based, found higher rates of dose escalation in patients initiating IFX (29% and 35% over 12 and 18 months, respectively) and ADA (8% and 10% over 12 and 18 months, respectively) compared to ETN (1% and 3% over 12 and 18 months, respectively) [11, 27]. While the results for IFX were similar to our study, we found lower rates of dose escalation for ADA and ETN. In DART II, Chastek, *et al*. [18] also found greater rates of dose escalation over 12 months in patients initiating IFX (50.3%) and ADA (9.5%) compared to ETN (1.7%) as did Cannon and colleagues [21], with 64% of IFX patients and 16% of ADA patients experiencing dose escalation compared to 2% of ETN patients. The Chastek and Cannon studies had higher dose escalation with ADA and IFX compared to our study; however, they were both US retrospective claims studies and used different definitions of dose escalation.

The proportion of patients with dose escalation was lower in our study than ranges previously reported, but maintained the same order of dose escalation, with the lowest proportion seen in patients initiating ETN (previous studies range from 1-10%), followed by ADA (range 8-34%), and the highest proportions among IFX-treated patients (range 17-64%) [12, 21-26]. This may be a result of our inclusion criterion requiring that patients remain on the anti-TNF for at least six months, thus potentially selecting patients who were better responders. Additionally, other studies have used different time periods and definitions of dose escalation that may explain the differences in results. Lack of dose escalation in ETN patients may be due to the weekly injections and because patients may not want to increase to more frequent administration. Also, the product monograph does not recommend doses higher than 50 mg per week [36]. Although ADA was increased from an injection every two weeks to a weekly injection in some patients, this does not follow the product monograph recommended dosing [37].

In the DART study, monotherapy at the index date was associated with increased risk of dose escalation [11]. Although we were unable to model escalation adjusting for potential confounders, our subgroup analysis did not detect any association between dose escalation and monotherapy, or any other patient characteristics. Monotherapy was more prevalent in our study in those treated with IFX compared to the DART study (25% in our study versus 9%), but less prevalent for ETN (28% versus 45%) and ADA (17% versus 26%) [27]. Real-world data from registries suggests that approximately 30% of patients on biologics (anti-TNF and non-anti-TNF blockers) are taking them as monotherapy, despite being frequently prescribed concomitant DMARDs [38, 39].

The number of patients with co-therapy (DMARD and/or glucocorticoid) intensification over 12 and 18 months was 26% and 32% of ADA patients, 16% and 16% of ETN patients, and 9% and 12% of IFX patients. In the DART study, similar rates of co-therapy intensification were seen over 12 and 18 months for IFX (7% and 12%) and ETN (6% and 16%), but intensification was less common for ADA (8% and 18%) [11, 27]. The lower number of ADA patients with co-therapy intensification in the DART study could be explained by the higher proportion of patients having their anti-TNF dose increased relative to the results presented here.

ADA patients were more likely to switch to another biologic compared to ETN patients. By 18 months, 22% of ADA patients had switched to another biologic compared with 6% of ETN patients and 4% of IFX patients.

Over 12 and 18 months the total cost of therapy was higher in patients who initiated IFX versus ETN, mostly due to the higher IV infusion costs for IFX. Over 18 months, ADA patients had a lower total cost compared to ETN, mostly due to the lower cost of the index anti-TNF, which may partially be due to 7% of patients discontinuing ADA and not switching to a new biologic compared to 2% of ETN patients. Dose de-escalation was observed in 3 patients who initiated ETN (1 patient over the first year and 2 patients between 12-18 months), 1 patient who initiated ADA (1 patient between 12-18 months), and 0 patients who initiated IFX. The cost difference between ADA and ETN was no longer statistically significant after adjusting for potential confounders. These findings are consistent with other studies that have documented higher costs in patients who initiated IFX compared to subcutaneous anti-TNFs [18, 21, 25, 40]. To evaluate how costs changed after the first year, monthly costs were calculated. The total monthly costs in the ETN cohort were the same over months 1-12 and months 13-18 ($1,860), while the monthly costs decreased over the last 6 months compared to the first 12 months in the ADA ($1,682 compared to $1,830) and IFX ($1,896 compared to $2,246) cohorts. The lower cost in the IFX group over months 13-18 was partly due to the loading dose during the first year, while the lower cost for ADA was partly due to patients who discontinued and did not switch to a new biologic. Total costs were higher for dose escalators than non-escalators, which match the results found in other studies [11, 12, 23, 41].

To our knowledge, this is the first study to assess dose escalation and costs in a real-world setting in Canada; however, we acknowledge certain limitations to this study. This study suffered from smaller than expected sample sizes, particularly over 18 months, leading to the inability to see therapy progression after 12 months for the subset of patients not included in the 18-month analysis. Results longer than 18 months would have been informative given the chronic nature of the disease. Reasons for choice of index anti-TNF were not collected and there may have been patient characteristics that influenced response to therapy. We were not able to examine potential confounders related to dose escalation. Since medical resources from other providers were not documented in specialist charts, broader costs of care were not included. Clinical measures of effectiveness were not consistently found in charts, and thus, not included. Lastly, newer biologics golimumab and certolizumab pegol were not included.

## CONCLUSION

This study demonstrates that physicians are more likely to escalate the dose of IFX, but optimize co-therapy with ADA and ETN. Patients treated with ETN had no dose escalation and were less likely to have DMARD and/or glucocorticoid intensification or switch to another biologic than patients initiating ADA. Additionally, patients initiating ETN had lower total (drug and drug-related) costs over 12 and 18 months compared to IFX-treated patients, and no difference compared to ADA patients when adjusted for potential confounders. Finally, patients with dose escalation had higher costs compared to those with no escalation.

## Data Sharing

The data contained in our database contains proprietary elements owned by Optum and, therefore, cannot be broadly disclosed or made publicly available at this time. The disclosure of this data to third party clients assumes certain data security and privacy protocols are in place and that the third party client has executed our standard license agreement which includes restrictive covenants governing the use of the data.

## Figures and Tables

*p<0.05 comparing Etanercept to Infliximab or Etanercept to Adalimumab ^1^Drug costs included anti-TNFs, switched biologics, DMARDs, and steroids; Drug-related costs included healthcare professional fees for IV infusions and IM or IA injections.

**Table 1:** Baseline demographic and clinical characteristics.

**Baseline Characteristics**	**Total** **(n=314)**	**ETN** **(n=156)**	**IFX** **(n=32)**	**ETN VS. IFX** **P-Value**	**ADA** **(n=126)**	**ETN VS. ADA ** **P-value**
**Age (years), mean (SD)**	56.3 (12.6)	55.5 (12.1)	59.4 (14.8)	0.111	56.6 (12.6)	0.458
Min, median, max	21.0, 56.0, 90.0	21.0, 54.5, 84.0	24.0, 59.5, 82.0		25.0, 56.5, 90.0	
**Female, n (%)**	241 (76.8)	122 (78.2)	26 (81.3)	0.816	93 (73.8)	0.402
**RA duration (years), mean (SD)**	9.0 (4.9)	9.3 (4.7)	8.1 (2.6)	0.124	8.9 (5.5)	0.615
**Quan-Charlson comorbidity score^1^, mean (SD)**	0.1 (0.5)	0.1 (0.5)	0.2 (0.6)	0.344	0.1 (0.5)	0.870
**Prior 3-month DMARD use, n (%)**						
None	79 (25.2)	47 (30.1)	8 (25.0)	0.672	24 (19.1)	0.039
One	95 (30.3)	46 (29.5)	14 (43.8)	0.145	35 (27.8)	0.792
More than one	140 (44.6)	63 (40.4)	10 (31.3)	0.427	67 (53.2)	0.041
**Initiation of anti-TNF, n (%)**						
Monotherapy	72 (22.9)	43 (27.6)	8 (25.0)	0.831	21 (16.7)	0.033
Concomitant DMARD	242 (77.1)	113 (72.4)	24 (75.0)	0.831	105 (83.3)	0.033
**Medication insurance, n (%)**						
Government	159 (50.6)	78 (50.0)	15 (46.9)	0.847	66 (52.4)	0.720
Private	125 (39.8)	60 (38.5)	15 (46.9)	0.430	50 (39.7)	0.902
None	12 (3.8)	8 (5.1)	1 (3.1)	1.000	3 (2.4)	0.356
Not government, but unknown if private or none	4 (1.3)	1 (0.6)	0 (0.00)	1.000	3 (2.4)	0.328
Unknown	14 (4.5)	9 (5.8)	1 (3.1)	1.000	4 (3.2)	0.396

^1^The updated Quan-Charlson comorbidity index score is based on 12 conditions. ETN=Etanercept, IFX=Infliximab, ADA=Adalimumab

**Table 2:** Dose escalation, co-therapy intensification, discontinuation, and dose de-escalation over 12 and 18 months.

	**ETN** **n=156** **n (%)**	**IFX** **n=32** **n (%)**	**ETN VS. IFX** **P-value**	**ADA** **n=126** **n (%)**	**ETN VS. ADA** **P-value**
**12 months after anti-TNF initiation**					
Dose escalation	0 (0.0)	12 (37.5)	<0.001	2 (1.6)	0.199
Dose escalation and/or DMARD intensification	17 (10.9)	13 (40.6)	<0.001	18 (14.3)	0.468
Dose escalation and/or DMARD and/or glucocorticoid intensification	25 (16.0)	15 (46.9)	<0.001	35 (27.8)	0.019
Discontinue anti-TNF and switch to another biologic	7 (4.5)	1 (3.1)	1.000	12 (9.5)	0.101
Discontinue anti-TNF but no switch	6 (3.8)	0 (0.0)	0.592	6 (4.8)	0.772
Dose de-escalation	1 (0.6)	0 (0.0)	1.000	0 (0.0)	1.000
**18 months after anti-TNF initiation**	**n=102**	**n=25**		**n=90**	
Dose escalation	0 (0.0)	8 (32.0)	<0.001	2 (2.2)	0.218
Dose escalation and/or DMARD intensification	11 (10.8)	10 (40.0)	0.001	17 (18.9)	0.151
Dose escalation and/or DMARD and/or glucocorticoid intensification	16 (15.7)	11 (44.0)	0.005	31 (34.4)	0.004
Discontinue anti-TNF and switch to another biologic	6 (5.9)	1 (4.0)	1.000	20 (22.2)	0.001
Discontinue anti-TNF but no switch	2 (2.0)	0 (0.0)	1.000	6 (6.7)	0.150
Dose de-escalation	2 (2.0)	0 (0.0)	1.000	1 (1.1)	1.000

ETN=Etanercept, IFX=Infliximab, ADA=Adalimumab

**Table 3:** Mean total costs (2014/2015 Canadian Dollars) per patient by dose escalation and no dose escalation over 12 and 18 months.

		**Total Costs^1^**					
**Cohort**		**No Dose Escalation over 12 Months** **(n=300)**	**Dose Escalation over 12 Months** **(n=14)**	**P-value**	**No Dose** **Escalation** **over 18** **Months** **(n=207)**	**Dose Escalation over 18 Months** **(n=10)**	**P-value**
Etanercept	N	156	0		102	0	
	Mean (SD)	$22,315 ($2,192)	-	-	$33,478 ($3,636)	-	-
Infliximab	N	20	12		17	8	
	Mean (SD)	$23,270 ($5,247)	$33,075 ($4,196)	<0.001	$33,163 ($8,822)	$49,289 ($7,661)	<0.001
Adalimumab	N	124	2		88	2	
	Mean (SD)	$21,849 ($1,621)	$29,055 ($4,782)	0.279	$31,754 ($3,681)	$45,353 ($1,761)	<0.001

^1^Total cost included drug costs and drug-related costs.

## LIST OF ABBREVIATIONS

ADA: = Adalimumab
CI: = Confidence interval
DART study: = Drug utilization and dosing patterns Assessment: A Retrospective observational study of subjects Treated for rheumatoid arthritis
DF: = Degrees of freedom
DMARD: = Disease-modifying antirheumatic drugs
ETN: = Etanercept
GLM: = Generalized linear model
IA: = Intra-articular
IFX: = Infliximab
IM: = Intramuscular
IV: = Intravenous
RA: = Rheumatoid arthritis
SD: = Standard deviation
TNF: = Tumor necrosis factor
