# Supplementary material for: Dose Escalation and Co-therapy Intensification Between Etanercept, Adalimumab, and Infliximab: The CADURA Study
Source: Open Rheumatol J. 2017 Oct 24;11:123–35. doi: 10.2174/1874312901711010123 (PMC5744265; doi:10.2174/1874312901711010123)
Supplement: Supplementary file 1 [file TORJ-11-123_SD1.pdf]

*Send Orders for Reprints to reprints@benthamscience.ae*



The Open Rheumatology Journal

Supplementary Material

Content list available at: www.benthamopen.com/TORJ/

DOI: 10.2174/1874312901711010123

RESEARCH ARTICLE

# Dose Escalation and Co-therapy Intensification Between Etanercept, Adalimumab, and Infliximab: The CADURA Study

Carter Thorne[1], Gilles Boire[2], Andrew Chow[3], Kirsten Garces[4], Fang Liu[5], Melanie Poulin-Costello[4], Valery Walker[5,*] and Boulos Haraoui[6]

[1]*The Arthritis Program Research Group, Southlake Regional Health Centre, c/o 43 Lundy's Lane, Newmarket, ON, L3Y 3R7, Canada*
[2]*Centre hospitalier universitaire de Sherbrooke (CIUSSS de l'Estrie-CHUS), Université de Sherbrooke, Sherbrooke, QC, Canada*
[3]*Credit Valley Rheumatology, Mississauga, ON, Canada*
[4]*Amgen Canada Inc., Mississauga, ON, Canada*
[5]*Optum, 5500 North Service Road, Suite 501, Burlington, ON, L7L 6W6, Canada*
[6]*Institut de Rhumatologie de Montreal, Montreal, QC, Canada*

## APPENDIX A

**Supplementary Table A. Generalized linear model base case and sensitivity analysis cost ratios for total cost over 12 months.**

| | Total Cost over 12-month Follow-up[1] | | | | Total Cost over 12-month Follow-up Sensitivity Analysis[2] | | | |
|---|---|---|---|---|---|---|---|---|
| **Independent Variables** | **Cost Ratio** | **Lower 95% CI** | **Upper 95% CI** | **P-value** | **Cost Ratio** | **Lower 95% CI** | **Upper 95% CI** | **P-value** |
| **Index Medication** | | | | | | | | |
| Etanercept | ref. | – | – | – | ref. | – | – | – |
| Adalimumab | 0.988 | 0.958 | 1.018 | 0.413 | 0.978 | 0.951 | 1.006 | 0.125 |
| Infliximab | 1.240 | 1.181 | 1.302 | <0.001 | 1.214 | 1.159 | 1.271 | <0.001 |
| **Charlson Comorbidity Score (continuous)** | 0.975 | 0.943 | 1.008 | 0.137 | 0.972 | 0.946 | 1.000 | 0.051 |
| **RA Duration (in years)** | | | | | | | | |
| 0-10 | ref. | – | – | – | ref. | – | – | – |
| 11-15 | 1.030 | 0.992 | 1.070 | 0.122 | 1.027 | 0.984 | 1.073 | 0.226 |
| 16+ | 1.024 | 0.976 | 1.073 | 0.335 | 1.008 | 0.954 | 1.066 | 0.772 |
| **Age (continuous)** | 1.000 | 0.999 | 1.002 | 0.445 | 1.000 | 0.999 | 1.001 | 0.770 |
| **Gender** | | | | | | | | |
| Male | 0.994 | 0.961 | 1.027 | 0.706 | 1.000 | 0.969 | 1.031 | 0.976 |
| Female | ref. | – | – | – | ref. | – | – | – |
| **Medication Insurance at Index Date** | | | | | | | | |
| Government | ref. | – | – | – | ref. | – | – | – |
| Private | 1.012 | 0.980 | 1.045 | 0.471 | 1.003 | 0.972 | 1.034 | 0.868 |
| None | 1.001 | 0.905 | 1.106 | 0.989 | 0.899 | 0.836 | 0.967 | 0.004 |
| Not government but unknown if private or none | 1.018 | 0.880 | 1.178 | 0.813 | 1.008 | 0.893 | 1.139 | 0.895 |
| Unknown | 1.029 | 0.950 | 1.115 | 0.485 | 1.009 | 0.942 | 1.081 | 0.789 |
| **Prior 3-month DMARD Use** | | | | | | | | |
| One | 1.021 | 0.951 | 1.097 | 0.568 | 1.032 | 0.974 | 1.093 | 0.283 |

1874-3129/17 2017 Bentham Open

*Suppl. Table 1 contd.....*

| Independent Variables | Total Cost over 12-month Follow-up[1] | | | | Total Cost over 12-month Follow-up Sensitivity Analysis[2] | | | |
|---|---|---|---|---|---|---|---|---|
| | Cost Ratio | Lower 95% CI | Upper 95% CI | P-value | Cost Ratio | Lower 95% CI | Upper 95% CI | P-value |
| More than one | 1.071 | 0.996 | 1.151 | 0.063 | 1.082 | 1.022 | 1.145 | 0.007 |
| None | ref. | – | – | – | ref. | – | – | – |
| **Initiation of Anti-TNF** | | | | | | | | |
| Monotherapy | 1.022 | 0.955 | 1.094 | 0.532 | 1.043 | 0.984 | 1.105 | 0.157 |
| Concomitant DMARD | ref. | – | – | – | ref. | – | – | – |

[1] Observations read=314, observations used=183, Pearson chi-square=1.541, DF=168

[2] Includes patients with RA duration missing, observations read=314, observations used=314, Pearson chi-square=4.190, DF=299

## APPENDIX B

**Supplementary Table B. Generalized linear model base case and sensitivity analysis cost ratios for total cost over 18 months.**

| Independent Variables | Total Cost over 18-month Follow-up[1] | | | | Total Cost over 18-month Follow-up Sensitivity Analysis[2] | | | |
|---|---|---|---|---|---|---|---|---|
| | Cost Ratio | Lower 95% CI | Upper 95% CI | P-value | Cost Ratio | Lower 95% CI | Upper 95% CI | P-value |
| **Index Medication** | | | | | | | | |
| Etanercept | ref. | – | – | – | ref. | – | – | – |
| Adalimumab | 0.965 | 0.927 | 1.006 | 0.090 | 0.958 | 0.917 | 1.002 | 0.059 |
| Infliximab | 1.197 | 1.125 | 1.275 | <0.001 | 1.160 | 1.082 | 1.243 | <0.001 |
| **Charlson Comorbidity Score (continuous)** | 0.927 | 0.885 | 0.970 | 0.001 | 0.941 | 0.901 | 0.981 | 0.005 |
| **RA Duration (in years)** | | | | | | | | |
| 0-10 | ref. | – | – | – | ref. | – | – | – |
| 11-15 | 1.020 | 0.968 | 1.075 | 0.455 | 1.022 | 0.954 | 1.096 | 0.528 |
| 16+ | 1.002 | 0.944 | 1.064 | 0.941 | 0.978 | 0.901 | 1.062 | 0.595 |
| **Age (continuous)** | 1.000 | 0.999 | 1.002 | 0.885 | 0.999 | 0.997 | 1.001 | 0.289 |
| **Gender** | | | | | | | | |
| Male | 0.995 | 0.950 | 1.042 | 0.826 | 1.017 | 0.966 | 1.070 | 0.527 |
| Female | ref. | – | – | – | ref. | – | – | – |
| **Medication Insurance at Index Date** | | | | | | | | |
| Government | ref. | – | – | – | ref. | – | – | – |
| Private | 1.012 | 0.966 | 1.059 | 0.618 | 1.003 | 0.954 | 1.054 | 0.920 |
| None | 1.004 | 0.882 | 1.143 | 0.947 | 0.901 | 0.804 | 1.010 | 0.075 |
| Not government but unknown if private or none | 1.024 | 0.868 | 1.208 | 0.781 | 0.994 | 0.848 | 1.166 | 0.944 |
| Unknown | 1.051 | 0.955 | 1.157 | 0.306 | 1.019 | 0.927 | 1.119 | 0.701 |
| **Prior 3-month DMARD Use** | | | | | | | | |
| One | 1.003 | 0.916 | 1.098 | 0.949 | 1.065 | 0.977 | 1.162 | 0.151 |
| More than one | 1.034 | 0.942 | 1.135 | 0.485 | 1.093 | 1.002 | 1.192 | 0.045 |
| None | ref. | – | – | – | ref. | – | – | – |
| **Initiation of Anti-TNF** | | | | | | | | |
| Monotherapy | 0.991 | 0.910 | 1.079 | 0.838 | 1.064 | 0.977 | 1.159 | 0.156 |
| Concomitant DMARD | ref. | – | – | – | ref. | – | – | – |

[1]Observations read=217, observations used=124, Pearson chi-square=1.205, DF=109

[2]Includes patients with missing RA duration, observations read=217, observations used=217, Pearson chi-square=4.717, DF=202

## APPENDIX C

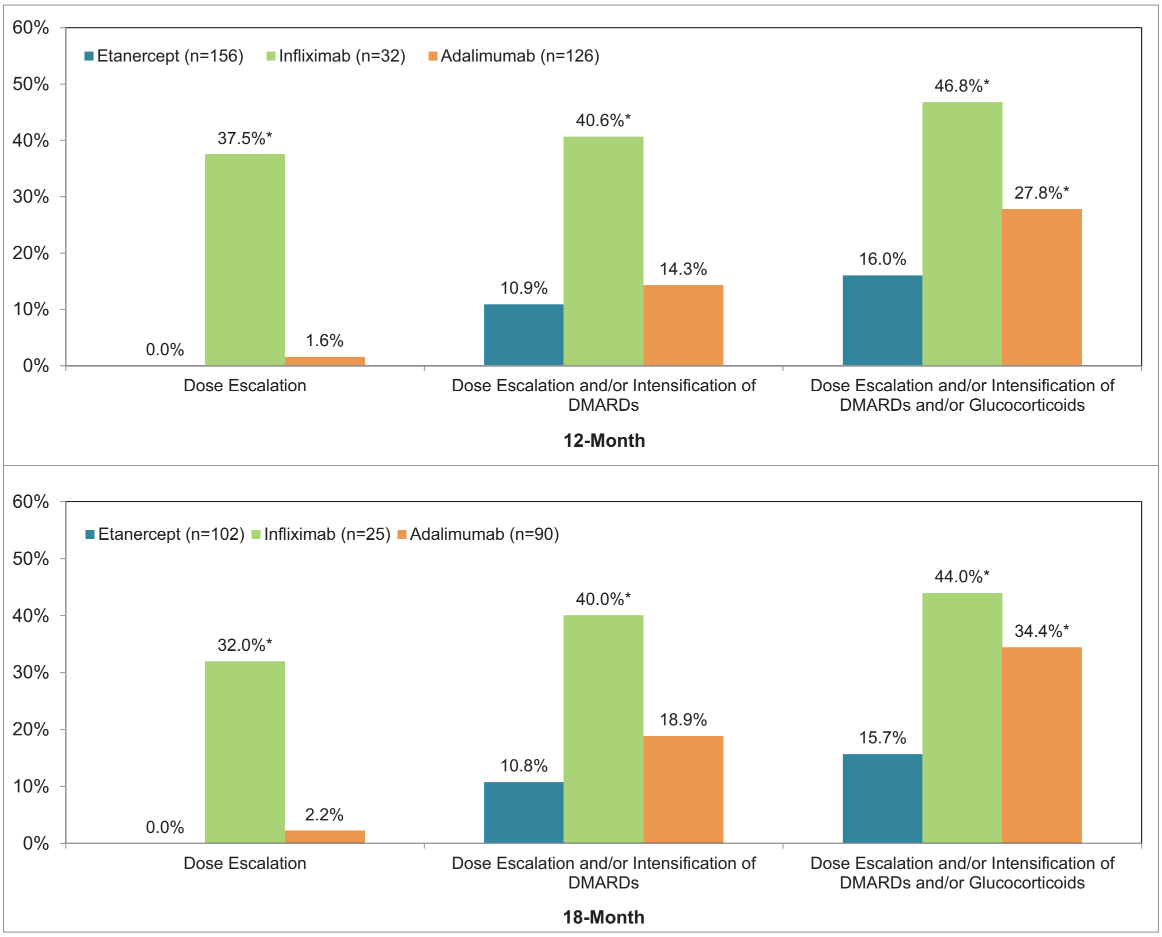


**Supplementary Fig. (A).** Percentage of Patients with anti-TNF dose escalation and DMARD and/or glucocorticoid intensification.

Abbreviations: DMARD=disease-modifying anti-rheumatic drug, TNF=tumor necrosis factor.

*$p<0.05$

© 2017 Thorne *et al.*

This is an open access article distributed under the terms of the Creative Commons Attribution 4.0 International Public License (CC-BY 4.0), a copy of which is available at: (https://creativecommons.org/licenses/by/4.0/legalcode). This license permits unrestricted use, distribution, and reproduction in any medium, provided the original author and source are credited.
